# Prednisone for patients with recurrent implantation failure: study protocol for a double-blind, multicenter, randomized, placebo-controlled trial

**DOI:** 10.1186/s13063-020-04630-6

**Published:** 2020-08-17

**Authors:** Yao Lu, Junhao Yan, Jiayin Liu, Jichun Tan, Yan Hong, Daimin Wei, Zi-jiang Chen, Yun Sun

**Affiliations:** 1 Center for Reproductive Medicine, Ren Ji Hospital, School of Medicine, Shanghai Jiao Tong University, Shanghai, 200135 China; 2 Shanghai Key Laboratory for Assisted Reproduction and Reproductive Genetics, 845 Lingshan Road, Pudong New District, Shanghai, 200135 China; 3 Center for Reproductive Medicine, Shandong Provincial Hospital Affiliated to Shandong University, Key Laboratory of Reproductive Endocrinology, Shandong University, Ministry of Education, and National Research Center for Assisted Reproductive Technology and Reproductive Genetics, Jinan, China; 4 Center of Clinical Reproductive Medicine, The First Affiliated Hospital of Nanjing Medical University, Nanjing, 210029 China; 5 Reproductive Medical Center, Obstetrics and Gynecology Department, Shengjing Hospital of China Medical University, Shenyang, China

**Keywords:** Prednisone, Recurrent implantation failure, Randomized controlled trial, Live birth

## Abstract

**Background:**

Recurrent implantation failure (RIF) brings great challenges to clinicians and causes deep frustration to patients. Previous data has suggested that prednisone may play a promising role in the establishment of pregnancy and help improve the pregnancy outcome in women with RIF. But there is insufficient evidence from randomized clinical trials that had adequate power to determine if prednisone can enhance live births as the primary outcome.

**Methods/design:**

This trial is a prospective, multicenter, randomized, double-blind, placebo-controlled clinical trial (1:1 ratio of prednisone versus placebo). Infertile patients with RIF who intend to undergo frozen-thawed embryo transfer (FET) after in vitro fertilization (IVF) or intracytoplasmic sperm injection (ICSI) or pre-implantation genetic testing for aneuploidy (PGT-A) will be enrolled and randomly assigned to two parallel groups. Participants will be given the treatment of prednisone or placebo from the start of endometrial preparation till the end of the first trimester of pregnancy if pregnant. The primary outcome is live birth rate.

**Discussion:**

The results of this study will provide evidence for the effect of prednisone on pregnancy outcomes in patients with RIF.

**Trial registration:**

Chinese Clinical Trial Registry, ChiCTR1800018783. Registered on 9 October 2018.

## Background

Nowadays, infertility affects 8–12% of couples at reproductive age and has become a global problem [1]. In vitro fertilization (IVF) is widely used and well received in couples with reproductive difficulties. However, despite the optimal use of reproductive technologies (such as controlled ovarian hyper-stimulation, assisted hatching, pre-implantation genetic testing, and frozen embryo transfer), implantation remains a rate-limiting step in IVF treatment. Implantation rate is reported to be about 25% when cleavage embryos are transferred and 40% when blastocysts are transferred, which indicates that many couples would remain infertile after multiple attempts at embryo transfer [2].

Recurrent implantation failure (RIF) refers to the clinical condition of failing to achieve a clinical pregnancy after several embryo transfers, which brings great challenges to clinicians and causes deep frustration to patients [3]. The prevalence of RIF varies from 10 to 20% and is difficult to estimate, due to the condition that there is yet no universally accepted consensus on the definition of RIF [2, 4, 5]. Failure of implantation can be attributed to embryo quality, endometrial receptivity, or both. While poor embryo quality is thought to be responsible for 30–50% of implantation failures, decreased endometrial receptivity is responsible for approximately two thirds of these failures [6, 7]. Thus, many interventions aiming at overcoming decreased endometrial receptivity have been proposed to improve pregnancy outcomes in women with RIF, but only a few are evidence-based [8, 9].

Prednisone is a common immunomodulatory agent, which can exert a range of positive effects on the treatment of autoimmune disorders as well as the establishment of early pregnancy [1, 10]. Studies have shown that prednisone could not only suppress uterine NK cells cytotoxicity and cytokine secretion in pre-implantation endometrium, but also stimulate the secretion of human chorionic gonadotropin (hCG) and promote proliferation and invasion of trophoblast [1, 6], suggesting that prednisone may have a considerable impact on embryo implantation and IVF outcomes. Prednisone is also believed to have minimal side effects [11], because only about 10% of the active substance will reach the fetus [12–14].

However, limited clinical trials have focused on the effect of prednisone on pregnancy outcomes. Also, the trials were either small-sized or non-randomized studies or with combined treatment regimens, which were insufficiently powered to draw a conclusion. Therefore, multiple researchers and clinicians have called for a full-scale and well-designed randomized controlled trial (RCT) to clarify whether prednisone could improve pregnancy outcomes in women with RIF [15].

## Methods/design

### Design and setting

**Fig. 1:**
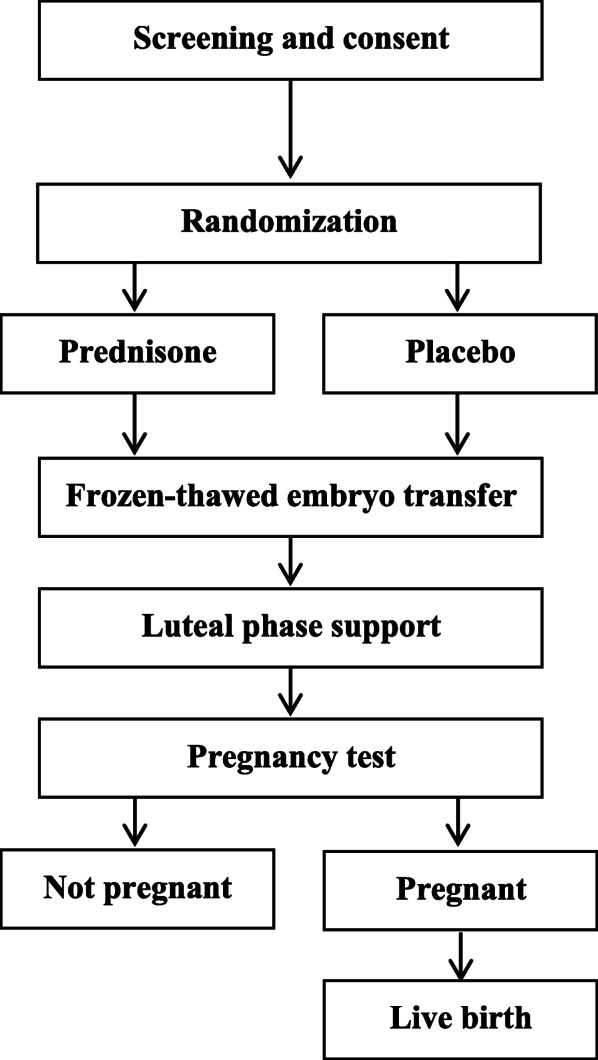
Flowchart of the study design

This is a prospective, multicenter, randomized, double-blind, placebo-controlled clinical trial to evaluate whether the administration of prednisone could improve the live birth rate in patients with RIF. Eligible patients will be randomly assigned to the prednisone group or placebo group with a 1:1 ratio. A flowchart of the study design is illustrated in Fig. 1.

Patients will be recruited from 8 hospitals in China. The study is conducted in accordance with the Declaration of Helsinki and Good Clinical Practice guidelines. The study protocol has been approved by the ethics committees at all hospitals. The trial was registered at the Chinese Clinical Trial Registry (ChiCTR1800018783; http://www.chictr.org.cn/edit.aspx?pid=31155&htm=4).

### Eligibility criteria

#### Inclusion criteria

The inclusion criteria are as follows:

1. Infertile women with a history of RIF, which refers to the failure to achieve a clinical pregnancy under one of the following conditions with all embryos transferred being of good quality (criteria of good-quality embryos are shown in Table 1):
  - Three or more embryo transfer cycles;
  - Two embryo transfer cycles where the cumulative number of transferred embryos was no less than three;
2. Women who intend to undergo frozen-thawed embryo transfer (FET) after IVF or intracytoplasmic sperm injection (ICSI) or pre-implantation genetic testing for aneuploidy (PGT-A);
3. Women under the age of 38 when oocytes were retrieved;
4. Women who have at least 1 good-quality blastocyst or 2 good-quality day-3 embryos for transfer
5. Be capable of giving informed consent.

**Table 1:** Criteria of good-quality embryo

Embryo	Scoring system	Criteria of good quality
Blastocyst	Gardner	≥ 4 BC
Cleavage (day 3)	Puissant	7–10 cell 3; 4 or compact
	Peter	7–10 cell I; II or compact

#### Exclusion criteria

The exclusion criteria are as follows:

1. Women who are currently receiving any corticosteroid or immunosuppression treatment, such as hydroxychloroquine, cyclosporine, and azathioprine. Two months of washout period will be required prior to screening for patients on these agents;
2. Women with known autoimmune diseases such as systemic lupus erythematosus (SLE), antiphospholipid syndrome, Sjogren’s syndrome, and scleroderma;
3. Women who have been diagnosed with diseases affecting the uterine cavity, such as uterine malformation and submucous fibroids;
4. Women or their partner with abnormal chromosome karyotype (not including chromosome polymorphisms);
5. Women who have experienced recurrent pregnancy loss, defined by two or more failed clinical pregnancies documented by ultrasonography or histopathologic examination;
6. Women with thin endometrium (< 6 mm before transfer);
7. Women with contraindications to corticosteroid;
8. Women with medical contraindications to assisted reproductive technology and/or pregnancy.

### Sample size

According to the meta-analysis published in 2013, the live birth rate was estimated to be 16.1% in women with 3 or more failed embryo transfer cycles [16]. In women with 2 or more failed embryo transfer cycles, the live birth rate varied from 15.5 to 29% [17–19]. It is reported that the combined administration of prednisone and low molecular weight heparin or aspirin can improve live birth rate by 10.8–14% [17, 20, 21]. In the present study, we plan to test the primary hypothesis of a difference of 10% in the live birth rate between the two randomization arms. In patients with RIF, we assume that the live birth rate will be 30% in the prednisone arm and 20% in the placebo arm.

For the sample size calculations, the two-sided significance level will be set at *α* = 0.05 and the statistical power will be calculated as 1 − *β* = 0.80. The ratio between groups will be 1:1. The minimum sample size will be 294 for each group, for a total of 588 participants. Taking into consideration a dropout rate of 15%, we expect to ultimately have a total of 692 enrollees.

### Randomization and blinding

All eligible subjects will be randomly assigned to one of the two study arms according to a computer-generated randomization sequence generated by the data coordinating center (DCC) with SAS software version 9.2 (SAS Institute, Cary, NC). The randomization will be stratified according to the stage of embryo (cleavage embryo or blastocyst). The randomization sequence will be kept strictly confidential by the DCC staff. Therefore, the researchers who are in charge of the enrollment have no access to the list. Study personnel are all blinded to the upcoming treatment group allocation.

The study medication (both prednisone and placebo) is manufactured by Xianju Pharmaceutical Co., Ltd. Except for the active ingredients, the rest of the excipient and the appearance and odor are exactly the same as prednisone. The packaging of study medication (both prednisone and placebo) is marked according to the randomization sequence. The packaging and tablets of prednisone and the placebo have the same appearance, which cannot be distinguished. Therefore, the participants and all research staff do not know the allocation until the end of the study. The quality of the placebo, such as contents and bacteria contaminations, was controlled rigorously according to the GMP standard.

### Screening

**Fig. 2:**
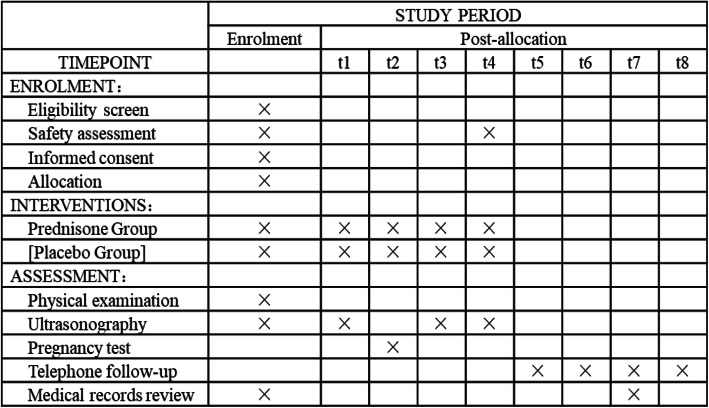
SPIRIT diagram for schedule of enrollment, interventions, and assessments

At the screening visit, patients who have been using corticosteroid or other immunosuppression treatment will be excluded. The trial and study plan will be declared to all participants, and eligible couples who are interested in participating will sign the written informed consent. All tests for preparation of IVF or ICSI or PGT-A have been done. Screening for possible causes of RIF such as parental chromosomal anomalies, uterine factors (hysteroscopy if necessary), hydrosalpinx, and immunological and thrombophilic factors including antinuclear antibody (ANA), anticardiolipin antibody (ACA), double-stranded DNA antibody (ds-DNA), β2-glycoprotein I antibody (β2GP1), and platelet aggregation test (PAGT), will be recommended. The standardized case report forms (CRFs) are completed to collect current medication status and previous medical history. A physical examination (height, body weight, waistline, hipline, blood pressure) is performed. A schedule of enrollment, interventions, and assessment is provided in the Standard Protocol Items: Recommendations for Interventional Trials (SPIRIT) figure (Fig. 2). The SPIRIT checklist is presented in Additional file 1.

Safety assessment includes liver function; renal function; CBC; fasting blood glucose; insulin; t1, day of embryo transfer (ET); t2, 2 weeks after ET; t3, 5 weeks after ET; t4, 10 weeks after ET; t5, 28 weeks of gestation; t6, 37 weeks of gestation; t7, delivery; and t8, 6 weeks after delivery.

### Interventions

A total of 692 eligible subjects will be enrolled and equally randomized into two parallel treatment arms:

A) Prednisone arm: oral prednisone arm 10 mg q.m. orally;
B) Placebo arm: oral prednisone-matched placebo q.m. orally.

After a recruitment period and randomization, the researchers will dispense the first bottle (100 tablets per bottle) of the corresponding drug (prednisone/placebo). Patients will be instructed to take two tablets for once a day orally in the morning, starting with the hormone replacement regimen for endometrial preparation. Participants will undergo the frozen-thawed embryo transfer (FET). If pregnancy is confirmed, the second bottle of corresponding drug will be dispensed on the day of pregnancy test and the medication will be continued till the end of the first trimester of pregnancy. If the failure of transfer or pregnancy loss occurred, the medication will be discontinued. The remaining tablets will be returned to researchers.

### Discontinuation criteria

Reasons for discontinuation of medication include, but are not limited to, the following: (1) participants who become pregnant spontaneously, (2) participants who have experienced serious complications or side effects, (3) participants who use prohibited drugs such as other glucocorticoids or immunosuppression drugs, (4) participants with thin endometrium (< 6 mm before transfer), and (5) participants who request to withdraw from the trial.

### Frozen-thawed embryo transfer

The endometrium is prepared with a hormone replacement cycle regimen. Estradiol valerate (Progynova, Delpharm Lille SAS, France) and/or estradiol tablets (Femoston, Abbott Biologicals B.V., the Netherlands) at a dose of 2–8 mg daily will be started on days 2–5 of the menstrual cycle or 28–35 days after long-acting GnRH-agonist suppression. When the thickness of the endometrium is enough (≥ 6 mm), luteal phase support will be added with vaginal progesterone gel (Crinone, Merck Serono) 90 mg daily and oral dydrogesterone (Duphaston, Abbott) 10 mg twice daily.

One blastocyst or two cleavage embryos will be transferred in each participant. All embryos transferred will be of good quality. The serum hCG test is performed after 2 weeks of transfer. If the patient is pregnant, luteal phase support will be continued until 8–12 weeks of gestation and follow-up will be continued to the end of pregnancy.

### Outcome and outcome assessments

#### Primary outcome

The primary outcome is live birth after frozen-thawed embryo transfer, defined as the delivery of any number of newborns at ≥ 28 weeks of gestation with signs of life.

#### Secondary outcomes

The secondary outcomes include biochemical pregnancy, clinical pregnancy, implantation, pregnancy loss, pregnancy and perinatal complication, birth weight, congenital anomalies, and other adverse events. Biochemical pregnancy is defined as serum β-hCG ≥ 10 mIU/mL measured 12–15 days after embryo transfer. Clinical pregnancy is defined as the detection of a gestational sac in the uterine cavity by a transvaginal ultrasound scan 35 days after embryo transfer. Implantation rate is calculated as the number of gestational sacs by a transvaginal ultrasound scan/number of embryos transferred.

#### Follow-up protocol

The first pregnancy follow-up will be at 12 weeks of gestation. The first-trimester pregnancy complications (including but not limited to miscarriage, ectopic pregnancy, hyperemesis gravidarum, or gestational trophoblastic disease) will be evaluated by reviewing medical records or by telephone. The study medication (prednisone/placebo) will be discontinued, and the date of drug withdrawal will be recorded.

The second pregnancy follow-up will be at 28 weeks’ gestation. The second-trimester pregnancy complications (including but not limited to abortion, prenatal test results, gestational diabetes, incompetent cervix, or premature rupture of membrane) will be followed up by telephone call.

The third pregnancy follow-up will be at 37 weeks’ gestation. The third-trimester pregnancy complications (including but not limited to preterm labor, intrauterine growth retardation, pre-eclampsia/eclampsia, premature rupture of membrane, placental abruption, or abnormality of amniotic fluid) will be followed up by telephone call.

The fourth follow-up time point will be at delivery. Participants will be required to notify investigators about the time of delivery. The delivery information (including gestational age, delivery mode, placenta abnormality, and/or delivery complications) and neonatal information (including gender, birth weight, birth defects) will be obtained by designed forms or by reviewing obstetric and neonatal medical records.

The fifth and final follow-up will be at 6 weeks after delivery. Postpartum information regarding complications of the mother (including but not limited to infection, postpartum depression, late postpartum hemorrhage) or the infant (including but not limited to neonatal jaundice, neonatal infection, neonatal respiratory distress syndrome, neonatal hospitalization, and neonatal death) will be followed up by telephone or by reviewing medical records.

During the follow-up period, adverse events and concomitant medications will be inquired and recorded every time. Participants who quit or are lost to follow-up will also be recorded.

#### Adverse events

Adverse events (AEs) refer to any untoward medical occurrences associated with the subject’s participation during the research period regardless of whether considered to be related to the study intervention or not. Serious adverse events (SAEs) are events that occur during the subject’s participation in research that meet any of the following criteria: death, life-threatening events, severe or persistent disability, requiring inpatient hospitalization or prolongation of existing hospitalization, neonatal death up to 6 weeks after delivery, congenital anomaly or birth defect, or any events deemed serious by the local principal investigator.

All AEs will be assessed and recorded in detail. All SAEs will be reported to the principal investigator in 5 days, and appropriate measures will be initiated. SAEs which are unintended and possibly associated with study interventions should be reported within 24 h. The ethics committee will determine whether the AE or SAE is likely to be associated with the study medication and whether it is necessary to break blinding codes.

### Data management

All of the investigators including physicians, nurses, and research assistants will attend a training workshop before the starting of the trial, to ensure the accuracy of outcome assessments and data collection. A protocol and standard operation procedures will be given to every investigator.

All data will be collected using a standard CRF and recorded in the electronic data capture system where participants’ personal information cannot be traced. The DCC is responsible for the monitoring tasks of the trial. The DCC and personnel of Ren Ji hospital will check the authenticity, accuracy, and integrity of the data from different sites regularly to ensure the quality of the collected data.

### Data analysis plan

The data will be analyzed by SPSS 21.0 (SPSS Inc., Chicago, IL, USA). Normally distributed continuous variables will be expressed as mean ± standard deviation with Student’s test for testing between-group differences. Non-normally distributed continuous variables will be expressed as medians and ranges with Wilcoxon’s rank-sum test for between-group differences. Categorical data will be described as frequency and percentage; differences in these measures will be assessed by the Pearson chi-square test, with Fisher’s exact test for expected frequencies less than 5. *P* < 0.05 will be considered significant.

The analysis will be conducted using intention-to-treat principles. The primary outcome, live birth rate, will be compared between two groups by the Pearson chi-square test. Secondary outcomes, such as rates of pregnancy and implantation, will be analyzed using the Pearson chi-square test. A per-protocol analysis will be performed according to the actual participants completing the entire trial. As a secondary analysis, we will fit a generalized linear mixed effect model with a logit link to compare the treatment arms with respect to the primary outcome of live birth, and binary secondary outcomes (e.g., rates of pregnancy and implantation), adjusting for factors such as randomization stratification of embryo stage, and other explanatory variables. A random intercept will be included to adjust for the correlation among patients within center.

## Discussion

This is a trial evaluating whether the administration of prednisone could improve live birth rate in patients with RIF under the age of 38 years who are undergoing FET after IVF or ICSI or PGT-A. We plan to enroll 692 subjects from 8 hospitals in China. The enrollment began in November 2018. At the time of manuscript preparation, more than 500 subjects have been enrolled. The result of this multicenter randomized trial will provide valid evidence for the effect of prednisone on pregnancy outcomes in women with RIF. We speculate that the administration of prednisone may improve live birth rate in patients with RIF.

As we all know, there is as yet no universally accepted definition of RIF [2]. Different descriptions were based on the number of previously failed cycles or the number of embryos transferred or a combination of both. Lukasz et al. did a systematic review of the definition of RIF used in human subjects and found enormous variability. The most commonly stated definitions were “three or more failed cycles” or “two or more failed cycles,” solely or in combination with the number of transferred embryos [4, 6, 22]. It is suggested to define RIF as the absence of implantation after two treatment cycles where the cumulative number of transferred embryos was no less than four for cleavage-stage embryos and no less than two for blastocysts, and all of the embryos transferred should be of good quality [4].

There are limited clinical trials assessing the efficacy of prednisone in patients with implantation failure. Only one randomized controlled trial was reported, which was conducted in 133 women who tested positive for ANA with a history of one IVF implantation failure, and the conclusion showed that combined treatment of prednisone 10 mg/day and aspirin 100 mg/day may improve reproductive outcomes in these patients [20]. A quasi-randomized, controlled trial conducted in 295 women with previously one or two failed ICSI attempts suggested a combination of prednisolone and low molecular weight heparin (LMWH) might have a positive effect on pregnancy and implantation rates [21]. A prospective pilot study confirmed that prednisolone was useful in patients with at least two previous IVF failures and serum antiovarian antibody [23]. However, a recent retrospective cohort study demonstrated that the administration of prednisolone with LMWH in women with RIF (two or more failed fresh IVF or ICSI cycles followed by embryo transfer) does not improve pregnancy outcomes [17].

Hence, the effect of prednisone in women with RIF remains controversial. Despite lacking of convincing evidence, prednisone is being used by more and more IVF centers and reproductive physicians all across the world. There is an urgent need for a well-designed, adequately powered RCT to prove the efficacy of prednisone in patients with RIF. This study is expected to provide a reliable answer.

## Trial status

The study enrollment began on 20 November 2018 and is expected to end in June 2020. The enrollment is ongoing at the time of manuscript submission. The trial protocol is version 3.0, dated 15 December 2018.

## Supplementary information

**Additional file 1.** SPIRIT 2013 Checklist: Recommended items to address in a clinical trial protocol and related documents.

## Data Availability

The datasets generated during the current study are available from the corresponding author on reasonable request.

## Abbreviations

AE: Adverse event
ACA: Anticardiolipin antibody
ANA: Antinuclear antibody
AOA: Antiovarian antibody
β2GP1: β2-glycoprotein I antibody
CBC: Complete blood count
CRF: Case report form
DCC: Data Coordinating Center
ds-DNA: Double-stranded DNA antibody
ET: Embryo transfer
FET: Frozen-thawed embryo transfer
hCG: Human chorionic gonadotrophin
ICSI: Intracytoplasmic sperm injection
IVF: In vitro fertilization
LMWH: Low molecular weight heparin
PAGT: Platelet aggregation test
PGT-A: Pre-implantation genetic testing for aneuploidy
RCT: Randomized controlled trial
RIF: Recurrent implantation failure
SAE: Serious adverse event
